# Comparable Protective Effects of Low- and High-Dose MK-7 on Bone Structure and Remodeling in a Rat Model of Osteoporosis Induced by Estrogen Deficiency and Glucocorticoid Exposure

**DOI:** 10.3390/nu18101605

**Published:** 2026-05-18

**Authors:** Hsin-Ju Chiang, Shu-Yuan Hsu, Steve Leu

**Affiliations:** 1 Graduate Institute of Clinical Medical Sciences, College of Medicine, Chang Gung University, Taoyuan 333323, Taiwan; haubay@gmail.com; 2 AN-AN Women and Children Clinic, Kaohsiung 807038, Taiwan; 3 Department of Anatomy, Graduate Institute of Biomedical Sciences, College of Medicine, Chang Gung University, Taoyuan 333323, Taiwan; 4 Institute for Translational Research in Biomedicine, Kaohsiung Chang Gung Memorial Hospital, Kaohsiung 833253, Taiwan; 5 Department of Biotechnology, College of Life Science, Kaohsiung Medical University, Kaohsiung 807378, Taiwan; 6 Department of Nutrition and Health Science, Fooyin University, Kaohsiung 831301, Taiwan

**Keywords:** menaquinone-7, osteoporosis, ovariectomy, glucocorticoid

## Abstract

**Background:** Estrogen deficiency and glucocorticoid exposure are major contributors to osteoporosis. Although menaquinone-7 (MK-7) exhibits osteoprotective effects, whether low-dose supplementation is comparable to high-dose treatment remains unclear. **Methods:** Female Sprague–Dawley rats were assigned to sham control (SC), ovariectomy plus dexamethasone (OVX+Dex), and OVX+Dex treated with low-dose MK-7 (100 μg/kg) or high-dose MK-7 (20 mg/kg). Bone microarchitecture, histopathology, and serum bone turnover markers were evaluated. **Results:** OVX+Dex induced marked deterioration of trabecular bone microarchitecture, characterized by reduced bone volume and structural disruption. These changes were accompanied by increased osteoclast activity (cathepsin K), decreased osteogenic and extracellular matrix–related markers (cbfa-1, osteonectin, and biglycan), and downregulation of osteoprotegerin, indicating a pronounced imbalance in bone remodeling. Serum analysis further revealed reduced estradiol levels and alterations in circulating bone turnover markers, consistent with a dysregulated high-turnover state. Both low- and high-dose MK-7 significantly improved bone microarchitecture, restored remodeling-related protein expression, and partially normalized serum calcium-regulating hormones and bone turnover markers (all *p* < 0.05), with no significant differences observed between doses. **Conclusions:** MK-7 attenuates osteoporosis by restoring the balance between bone resorption and formation. Notably, low-dose MK-7 provides protective effects comparable to high-dose treatment, supporting its potential clinical utility.

## 1. Introduction

Osteoporosis is the most prevalent metabolic bone disorder and a major global health concern [1,2,3]. Recent large-scale meta-analyses have estimated that the global prevalence of osteoporosis is approximately 18%, with a markedly higher prevalence in women (~23%) compared to men (~12%) [4]. The condition is characterized by reduced bone mineral density, deterioration of bone microarchitecture, and an increased risk of fragility fractures. These changes collectively contribute to substantial morbidity, mortality, and healthcare burden, particularly in aging populations [2].

Multiple risk factors contribute to the development of osteoporosis, among which estrogen deficiency and long-term glucocorticoid exposure are the two most prominent [5,6,7]. Glucocorticoids, widely prescribed for inflammatory and autoimmune diseases, as well as post-transplant immunosuppression, are well known to accelerate bone loss by disrupting the balance between bone formation and resorption, thereby increasing fracture risk [8,9]. Despite advances in pharmacological therapies, including antiresorptive and anabolic agents, the global burden of osteoporosis continues to rise [10,11,12]. Current treatments are often associated with limitations such as adverse effects, high cost, or suboptimal long-term adherence [13,14]. Therefore, safe and effective alternative strategies for both prevention and treatment remain urgently needed, particularly for high-risk populations such as postmenopausal women receiving glucocorticoid therapy.

Vitamin K2 has emerged as a potential modulator of bone metabolism. It plays a critical role in the γ-carboxylation of osteocalcin, thereby enhancing its calcium-binding capacity and contributing to bone mineralization [15,16]. Clinical and experimental studies have demonstrated that vitamin K2 supplementation can reduce bone turnover and improve bone strength, particularly in postmenopausal populations [17,18,19]. Among its isoforms, menaquinone-7 (MK-7) exhibits superior bioavailability and a longer half-life, making it a promising candidate for osteoporosis intervention [19,20].

However, most previous studies have primarily focused on bone mineral density or biochemical markers, while the effects of MK-7 on bone remodeling–related changes within the bone microenvironment remain incompletely characterized [19,20]. Given that the bone marrow niche plays an important role in regulating the balance between osteoblast and osteoclast activity, histopathological evaluation may provide additional insight into osteoporosis progression and treatment response.

In the present study, we hypothesized that MK-7 supplementation would attenuate osteoporosis induced by combined estrogen deficiency and glucocorticoid exposure by preserving trabecular bone microarchitecture and restoring the balance between bone resorption and bone formation. We further hypothesized that low-dose MK-7 would provide protective effects comparable to those of high-dose treatment. Accordingly, we aimed to investigate the osteoprotective effects of MK-7 in a rat model of combined ovariectomy- and glucocorticoid-induced osteoporosis through integrated assessment of bone microarchitecture, remodeling-related markers, and serum biochemical profiles.

## 2. Materials and Methods

### 2.1. Ethics

All animal experimental procedures were approved by the Institutional Animal Care and Use Committee of Kaohsiung Chang Gung Memorial Hospital (Approval No. 2013031402) and were conducted in accordance with the National Institutes of Health Guide for the Care and Use of Laboratory Animals. All methods are reported in accordance with the ARRIVE guidelines (https://arriveguidelines.org) for the reporting of animal experiments [21].

### 2.2. Animals and Experimental Groups

The combined ovariectomy and dexamethasone protocol was used to model osteoporosis associated with estrogen deficiency and chronic glucocorticoid exposure, thereby reflecting clinically relevant high-risk conditions. On day 0, pathogen-free, 10-week-old female Sprague–Dawley (SD) rats (weighing 325–350 g; Charles River Technology, BioLASCO Co., Ltd., Taipei, Taiwan) were randomly assigned to four groups (*n* = 6 per group): (1) sham control (SC; laparotomy only), (2) ovariectomy plus dexamethasone (OVX+Dex), (3) OVX+Dex with low-dose MK-7 supplementation (100 μg/kg in diet), and (4) OVX+Dex with high-dose MK-7 supplementation (20 mg/kg in diet). A total of 24 animals were used in this study. The sample size was based on previous studies using similar osteoporosis models. The individual animal was defined as the experimental unit. Animals were housed under identical conditions to minimize potential confounding effects. Animals with severe postoperative complications, unexpected illness, or death before the endpoint would have been excluded; however, no animals were excluded from the final analysis.

Bilateral ovariectomy was performed to induce estrogen deficiency. Dexamethasone (2.5 mg/kg) was administered intramuscularly once every two weeks, starting two weeks after surgery and continuing until day 120 to mimic long-term glucocorticoid exposure. MK-7 (menaquinone-7; GeneFerm Biotechnology, Tainan, Taiwan), derived from fermented soybean (natto), was dissolved in soybean oil and incorporated into standard rodent chow. MK-7 supplementation was initiated at day 30 after ovariectomy and continued for 90 days.

All animal experiments were conducted in an Association for Assessment and Accreditation of Laboratory Animal Care International (AAALAC)-accredited facility under controlled environmental conditions (24 °C; 12 h light/12 h dark cycle). Animals were regularly monitored for health status, postoperative recovery, pain/distress, and adverse events. Perioperative care was provided to minimize pain and distress. Predefined humane endpoints included severe weight loss, marked distress, impaired mobility, wound complications, or inability to access food or water. No animals reached these endpoints or required early euthanasia during the study. At the designated experimental endpoint, animals were deeply anesthetized with inhalational isoflurane and euthanized by isoflurane overdose followed by exsanguination. Death was confirmed by the absence of spontaneous respiration, heartbeat, and reflex responses prior to tissue harvesting. Femurs were then immediately collected for subsequent immunohistochemical analyses.

### 2.3. Micro-Computed Tomography Analysis of Bone Microarchitecture

Bone microarchitecture was evaluated at day 120 using a high-resolution micro-computed tomography (micro-CT) system (Skyscan 1174, Bruker micro-CT, Kontich, Belgium) under inhalational anesthesia with 2.0% isoflurane. The distal femoral metaphysis was selected as the region of interest (ROI). Bone volume fraction (BV/TV) was quantified to assess trabecular bone structure and trabecular number. Image reconstruction and analysis were performed using the manufacturer’s software according to standardized protocols.

### 2.4. Immunohistochemical Staining and Quantification

Immunohistochemical staining was performed as described in our previous studies with minor modifications [22]. Briefly, femoral specimens were fixed in 10% neutral-buffered formalin and decalcified in EDTA solution prior to paraffin embedding. Serial sections (4–5 μm) were obtained from the distal femoral metaphysis. The paraffin-embedded sections were deparaffinized, rehydrated, and treated with 3% hydrogen peroxide for 30 min to block endogenous peroxidase activity, followed by incubation with a blocking reagent (Immuno-Block, BioSB, Santa Barbara, CA, USA) for 30 min at room temperature. Sections were then incubated with primary antibodies against Cbfa1 (1:200, Abcam, Cambridge, UK), biglycan (1:400, Abcam, Cambridge, UK), osteonectin (1:200, BioSS, Woburn, MA, USA), osteoprotegerin (1:100, Abbiotec, San Diego, CA, USA), and cathepsin K (1:200, Abcam, Cambridge, UK). Immunoreactivity was visualized using a standard DAB detection system, followed by hematoxylin counterstaining.

For quantification, a semi-quantitative scoring system was applied based on the percentage of positively stained cells in a blinded manner. The staining score was defined as follows: 0 = negative staining; 1 = <15%; 2 = 15–25%; 3 = 25–50%; 4 = 50–75%; and 5 = >75% positive cells per high-power field (HPF). For specimens with low expression levels (<15% positive cells), a modified semi-quantitative scoring approach was applied, in which the percentage of positive cells was recorded directly to provide higher resolution within this range. For each specimen, three sections were analyzed, and three randomly selected HPFs (×400) per section were evaluated. The mean value for each animal was calculated and used for statistical analysis. All evaluations were performed by a blinded observer.

### 2.5. Serum Biochemical Analysis

To evaluate systemic changes in mineral metabolism and bone turnover, blood samples were collected at the designated experimental endpoint. After anesthesia, whole blood was obtained via cardiac puncture and allowed to clot at room temperature, followed by centrifugation to obtain serum. Serum calcium levels were measured using a colorimetric assay kit based on the o-cresolphthalein complexone method (Sigma-Aldrich, St. Louis, MO, USA) according to the manufacturer’s protocols. Serum levels of estradiol (E_2_), parathyroid hormone (PTH), and osteocalcin were measured using commercially available enzyme-linked immunosorbent assay (ELISA) kits (R&D Systems, Minneapolis, MN, USA) according to the manufacturer’s instructions.

### 2.6. Statistical Analysis

Data are presented as mean ± standard deviation (SD). Normality and homogeneity of variance were assessed before statistical analysis. Differences among multiple groups were analyzed using one-way analysis of variance (ANOVA), followed by Tukey’s multiple comparisons post hoc test. Statistical analyses were performed using Prism 11 software (GraphPad Software, La Jolla, CA, USA). A *p* value < 0.05 was considered statistically significant.

## 3. Results

### 3.1. MK-7 Preserves Trabecular Bone Structure and Volume

**Figure 1:**
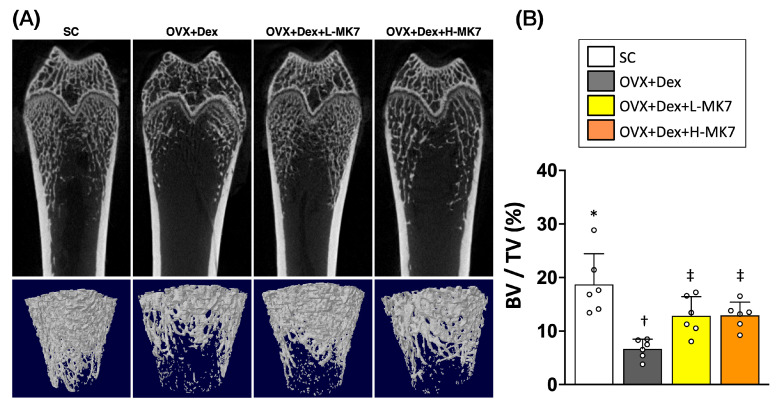
Effects of MK-7 supplementation on bone microarchitecture in ovariectomy- and glucocorticoid-induced osteoporosis. (**A**) Representative micro-computed tomography (micro-CT) images and corresponding three-dimensional reconstructions of trabecular bone in the distal femur from different experimental groups: sham control (SC), ovariectomy plus dexamethasone (OVX+Dex), OVX+Dex treated with low-dose MK-7 (L-MK7), and OVX+Dex treated with high-dose MK-7 (H-MK7). Marked deterioration of trabecular structure was observed in the OVX+Dex group compared with the SC group, whereas MK-7 supplementation partially preserved trabecular architecture. (**B**) Quantitative analysis of bone volume fraction (BV/TV, %). BV/TV was significantly reduced in the OVX+Dex group compared with the SC group, while both low- and high-dose MK-7 treatments significantly improved BV/TV. Data are presented as mean ± SD (*n* = 6 per group). Different symbols (*, †, ‡) indicate statistically significant differences among groups (*p* < 0.05).

As shown in Figure 1, the OVX+Dex group exhibited marked deterioration of trabecular microarchitecture compared with the sham control (SC) group, characterized by sparse, fragmented, and disconnected trabecular networks. In contrast, both low-dose and high-dose MK-7 treatments noticeably preserved trabecular structure, with denser and more interconnected trabeculae observed compared with the OVX+Dex group (Figure 1A).

Consistent with these structural observations, quantitative analysis demonstrated that bone volume fraction (BV/TV) was significantly reduced in the OVX+Dex group relative to the SC group (Figure 1B). Importantly, MK-7 supplementation at both low and high doses significantly increased BV/TV compared with the OVX+Dex group, indicating a partial restoration of bone mass. Although the values did not fully return to those of the SC group, the improvement was evident in both treatment groups. These findings suggest that MK-7 effectively attenuates bone loss and preserves trabecular microarchitecture under conditions of ovariectomy- and glucocorticoid-induced osteoporosis.

### 3.2. MK-7 Attenuates Bone Resorption and Matrix Remodeling

**Figure 2:**
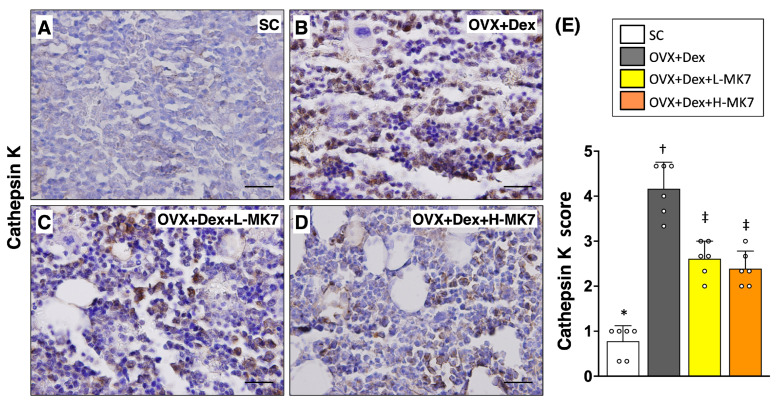
Effects of MK-7 supplementation on osteoclast-associated cathepsin K expression in bone tissue under ovariectomy and glucocorticoid-induced osteoporosis. (**A**–**D**) Representative immunohistochemical (IHC) staining of cathepsin K in femoral bone sections from different experimental groups: (**A**) sham control (SC), (**B**) ovariectomy plus dexamethasone (OVX+Dex), (**C**) OVX+Dex treated with low-dose MK-7 (L-MK7), and (**D**) OVX+Dex treated with high-dose MK-7 (H-MK7). Increased cathepsin K–positive cells (brown staining) were observed in the OVX+Dex group compared with the SC group. Nuclei were counterstained with hematoxylin (blue). Scale bars = 50 μm. (**E**) Semi-quantitative analysis of cathepsin K expression based on an IHC scoring system. Data are presented as mean ± SD (*n* = 6 per group). Different symbols (*, †, ‡) indicate statistically significant differences among groups (*p* < 0.05).

**Figure 3:**
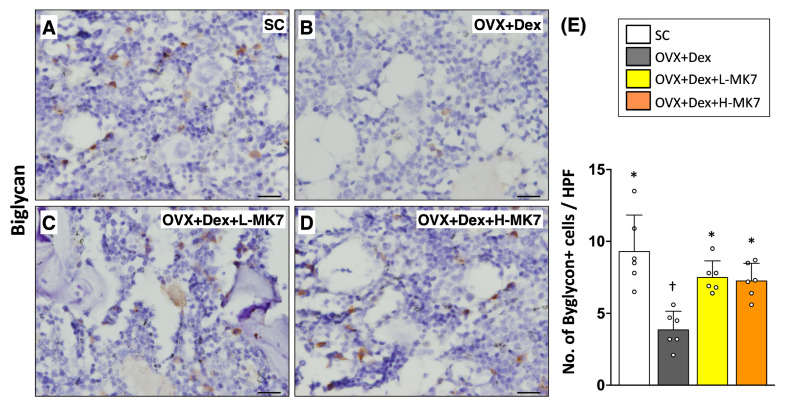
Effects of MK-7 supplementation on extracellular matrix–related biglycan expression in bone tissue under ovariectomy and glucocorticoid-induced osteoporosis. (**A**–**D**) Representative immunohistochemical (IHC) staining of biglycan in femoral bone sections from different experimental groups: (**A**) sham control (SC), (**B**) ovariectomy plus dexamethasone (OVX+Dex), (**C**) OVX+Dex treated with low-dose MK-7 (L-MK7), and (**D**) OVX+Dex treated with high-dose MK-7 (H-MK7). Reduced biglycan expression (brown staining) was observed in the OVX+Dex group compared with the SC group. Nuclei were counterstained with hematoxylin (blue). Scale bars = 50 μm. (**E**) Semi-quantitative analysis of biglycan expression based on an IHC scoring system. Data are presented as mean ± SD (*n* = 6 per group). Different symbols (*, †) indicate statistically significant differences among groups (*p* < 0.05).

Consistent with the structural analysis, immunohistochemical staining demonstrated that bone resorption-related activity was enhanced in the OVX+Dex group. The expression of cathepsin K was markedly increased compared with the sham control group (*p* < 0.05), indicating upregulation of osteoclast activity (Figure 2). In contrast, biglycan expression was significantly reduced in the OVX+Dex group, suggesting altered extracellular matrix remodeling under pathological conditions (Figure 3). Following MK-7 supplementation, cathepsin K expression was reduced, whereas biglycan expression was increased compared with the OVX+Dex group (all *p* < 0.05), but both did not return to sham control levels. No significant differences were observed between the low- and high-dose MK-7 groups.

### 3.3. MK-7 Restores Osteogenic Activity and Bone Remodeling Balance

**Figure 4:**
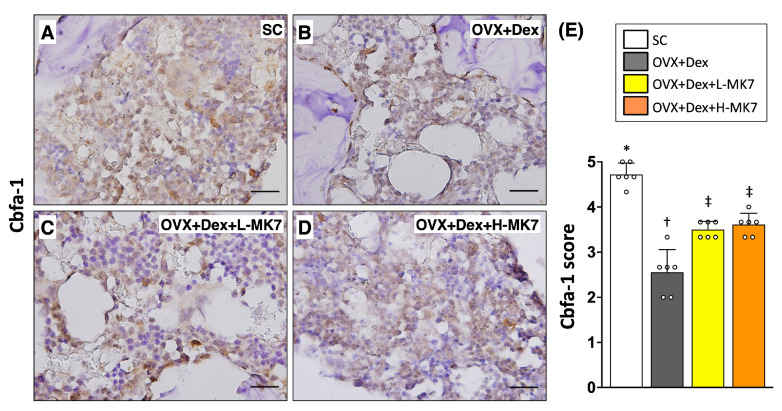
Effects of MK-7 supplementation on osteoblast-associated cbfa-1 expression in bone tissue under ovariectomy and glucocorticoid-induced osteoporosis. (**A**–**D**) Representative immunohistochemical (IHC) staining of cbfa-1 in femoral bone sections from different experimental groups: (**A**) sham control (SC), (**B**) ovariectomy plus dexamethasone (OVX+Dex), (**C**) OVX+Dex treated with low-dose MK-7 (L-MK7), and (**D**) OVX+Dex treated with high-dose MK-7 (H-MK7). Reduced cbfa-1–positive cells (brown staining) were observed in the OVX+Dex group compared with the SC group. Nuclei were counterstained with hematoxylin (blue). Scale bars = 50 μm. (**E**) Semi-quantitative analysis of cbfa-1 expression based on an IHC scoring system. Data are presented as mean ± SD (*n* = 6 per group). Different symbols (*, †, ‡) indicate statistically significant differences among groups (*p* < 0.05).

**Figure 5:**
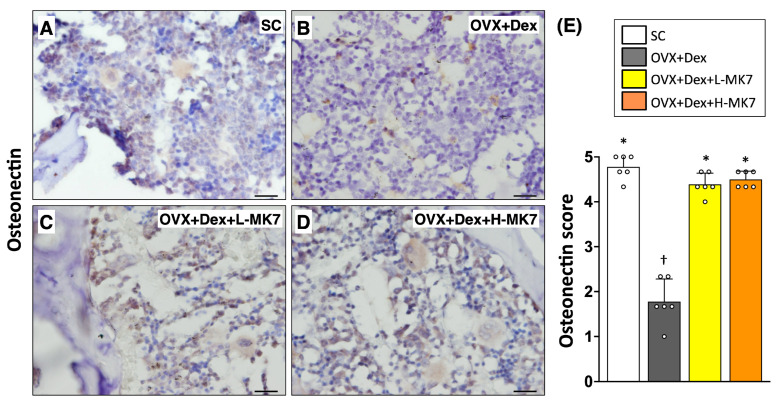
Effects of MK-7 supplementation on matrix-associated osteonectin expression in bone tissue under ovariectomy and glucocorticoid-induced osteoporosis. (**A**–**D**) Representative immunohistochemical (IHC) staining of osteonectin in femoral bone sections from different experimental groups: (**A**) sham control (SC), (**B**) ovariectomy plus dexamethasone (OVX+Dex), (**C**) OVX+Dex treated with low-dose MK-7 (L-MK7), and (**D**) OVX+Dex treated with high-dose MK-7 (H-MK7). Decreased osteonectin expression (brown staining) was observed in the OVX+Dex group compared with the SC group. Nuclei were counterstained with hematoxylin (blue). Scale bars = 50 μm. (**E**) Semi-quantitative analysis of osteonectin expression based on an IHC scoring system. Data are presented as mean ± SD (*n* = 6 per group). Different symbols (*, †) indicate statistically significant differences among groups (*p* < 0.05).

**Figure 6:**
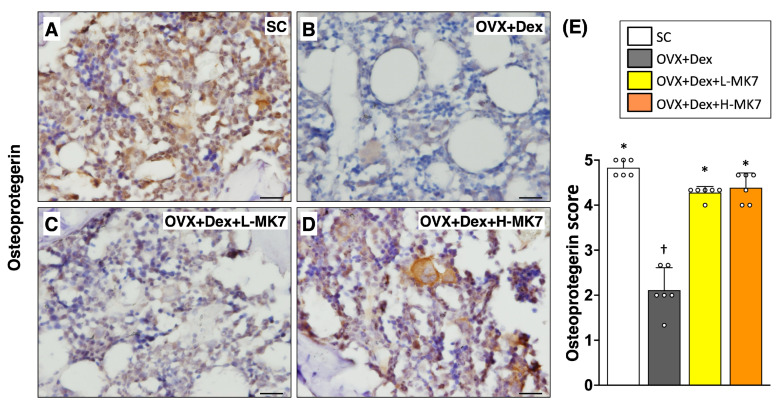
Effects of MK-7 supplementation on remodeling-related osteoprotegerin expression in bone tissue under ovariectomy and glucocorticoid-induced osteoporosis. (**A**–**D**) Representative immunohistochemical (IHC) staining of osteoprotegerin in femoral bone sections from different experimental groups: (**A**) sham control (SC), (**B**) ovariectomy plus dexamethasone (OVX+Dex), (**C**) OVX+Dex treated with low-dose MK-7 (L-MK7), and (**D**) OVX+Dex treated with high-dose MK-7 (H-MK7). Reduced osteoprotegerin expression (brown staining) was observed in the OVX+Dex group compared with the SC group. Nuclei were counterstained with hematoxylin (blue). Scale bars = 50 μm. (**E**) Semi-quantitative analysis of osteoprotegerin expression based on an IHC scoring system. Data are presented as mean ± SD (*n* = 6 per group). Different symbols (*, †) indicate statistically significant differences among groups (*p* < 0.05).

In contrast to the increased bone resorption, immunohistochemical staining showed that osteogenesis-related activity was suppressed in the OVX+Dex group. The expression of core-binding factor subunit alpha-1 (cbfa-1) was reduced compared with the sham control group (*p* < 0.05), indicating impaired osteoblastic differentiation (Figure 4). Similarly, osteonectin expression was decreased in the OVX+Dex group, reflecting reduced bone matrix formation (Figure 5). In addition, osteoprotegerin expression was also reduced compared with the sham control group (*p* < 0.05), suggesting disruption of bone remodeling balance (Figure 6). Following MK-7 supplementation, the expression of cbfa-1, osteonectin, and osteoprotegerin increased compared with the OVX+Dex group (all *p* < 0.05), but did not return to sham control levels. No significant differences were observed between the low- and high-dose MK-7 groups.

### 3.4. MK-7 Restores Serum Calcium-Regulating Hormones and Bone Turnover Markers

**Figure 7:**
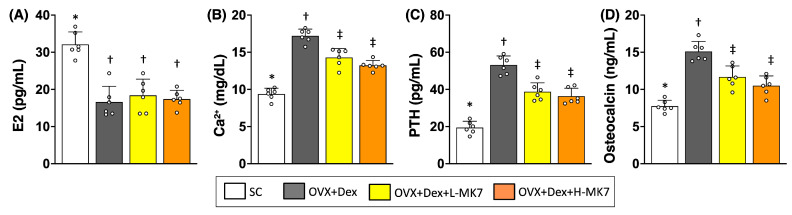
Effects of MK-7 supplementation on serum hormonal profiles and bone turnover markers under ovariectomy and glucocorticoid-induced osteoporosis. (**A**) Serum estradiol (E2) levels. (**B**) Serum calcium (Ca^2+^) levels. (**C**) Serum parathyroid hormone (PTH) levels. (**D**) Serum osteocalcin levels in different experimental groups: sham control (SC), ovariectomy plus dexamethasone (OVX+Dex), OVX+Dex treated with low-dose MK-7 (L-MK7), and OVX+Dex treated with high-dose MK-7 (H-MK7). Reduced serum E2 levels and increased Ca^2+^, PTH, and osteocalcin levels were observed in the OVX+Dex group compared with the SC group. MK-7 supplementation partially reversed these alterations, except for E2 levels. Data are presented as mean ± SD (*n* = 6 per group). Different symbols (*, †, ‡) indicate statistically significant differences among groups (*p* < 0.05).

In line with the structural and histological findings, serum biochemical analysis revealed marked alterations in systemic bone metabolism–related markers in the OVX+Dex group. Serum E2 levels were reduced compared with the sham control group (*p* < 0.05), confirming successful induction of estrogen deficiency (Figure 7). Concomitantly, circulating Ca^2+^, PTH, and osteocalcin levels were elevated, reflecting a dysregulated state of calcium homeostasis accompanied by increased bone turnover.

Following MK-7 supplementation, serum PTH, and osteocalcin levels decreased relative to the OVX+Dex group (all *p* < 0.05), indicating partial restoration of systemic bone remodeling-related regulation. Serum Ca^2+^ levels also declined after MK-7 treatment, although they remained higher than those in the sham control group. Notably, MK-7 supplementation did not restore serum E2 levels. Furthermore, no significant differences were observed between the low- and high-dose MK-7 groups across all measured parameters. Together, these findings support a role for MK-7 in modulating systemic bone metabolism under osteoporotic conditions.

## 4. Discussion

The present study demonstrates that MK-7 supplementation effectively attenuates bone loss and remodeling imbalance in a rat model of combined ovariectomy- and glucocorticoid-induced osteoporosis. Integrated analyses revealed that MK-7 preserved trabecular bone microarchitecture, suppressed osteoclast-associated activity, and restored osteogenic and extracellular matrix–related marker expression, accompanied by partial normalization of systemic bone metabolism–related profiles. Notably, both low- and high-dose MK-7 exerted comparable protective effects across structural, histological, and biochemical parameters. These findings suggest that MK-7 mitigates osteoporosis through coordinated regulation of bone remodeling processes and systemic metabolic homeostasis, with efficacy that appears to be independent of dose within the tested range.

The combined ovariectomy and glucocorticoid model used in the present study has been previously established as a clinically relevant approach to mimic complex osteoporotic conditions, particularly those associated with postmenopausal status and long-term glucocorticoid exposure [23,24,25]. Prior studies using this model have consistently demonstrated marked deterioration of bone microarchitecture and increased bone turnover [23,24,25]; however, most previous studies have primarily focused on bone mineral density and mRNA expression analyses, with comparatively limited histological evaluation [24,25]. In contrast, the present study extends these findings by providing a more integrated evaluation of bone remodeling, incorporating structural, histopathological, and systemic biochemical analyses.

Specifically, our results demonstrate that MK-7 not only preserves trabecular bone architecture but also exerts coordinated regulatory effects on osteoclast activity, osteogenic differentiation, and extracellular matrix remodeling, as evidenced by alterations in cathepsin K, cbfa-1, osteonectin, biglycan, and osteoprotegerin expression. These findings are consistent with previous reports suggesting that vitamin K2 contributes to bone formation and inhibits bone resorption through modulation of osteoblast and osteoclast function [26,27,28]. Furthermore, the parallel improvement observed in circulating markers, particularly osteocalcin, is consistent with the established role of vitamin K2 in regulating osteocalcin activation and bone mineralization, while the associated changes in Ca^2+^ and PTH may reflect secondary effects on systemic calcium homeostasis [29,30].

Notably, the present study demonstrates that low-dose MK-7 supplementation confers protective effects comparable to those of high-dose treatment across structural, histological, and biochemical parameters. This finding suggests that the osteoprotective actions of MK-7 may reach a functional threshold within the tested dose range, beyond which additional supplementation does not yield further measurable benefits. Such a response is consistent with the established role of vitamin K2 in facilitating γ-carboxylation-dependent processes, which may become saturated at relatively low concentrations [20]. From a clinical perspective, lower-dose supplementation may offer advantages in terms of safety, cost-effectiveness, and long-term adherence, particularly in populations requiring prolonged intervention, such as postmenopausal individuals or patients receiving chronic glucocorticoid therapy. Therefore, the comparable efficacy observed with low-dose MK-7 supports its potential use as a practical and accessible nutritional or adjunctive strategy for osteoporosis management.

To further address the translational relevance of the dosing regimen, we estimated the human equivalent dose (HED) of MK-7 based on body surface area normalization [31]. Using this approach, the rat-to-human Km ratio is 6/37, corresponding to a conversion factor of approximately 0.162. Accordingly, the low-dose MK-7 regimen used in the present study (100 μg/kg in rats) corresponds to an estimated HED of approximately 16.2 μg/kg, or about 970 μg/day for a 60 kg adult, whereas the high-dose regimen (20 mg/kg in rats) corresponds to approximately 3.24 mg/kg, or about 194 mg/day for a 60 kg adult. Notably, even the estimated HED of the low-dose regimen is higher than MK-7 doses commonly used in previous human MK-7 supplementation studies, such as 180–400 μg/day [20,32,33]. Therefore, these calculations should be interpreted as approximate translational estimates rather than direct clinical dosing recommendations. Importantly, the lack of additional benefit with high-dose MK-7 suggests that excessive dose escalation may not provide greater osteoprotective effects. This finding supports the need for future studies to define a lower and clinically feasible dosing range for osteoporosis prevention or adjunctive management.

This study has several limitations. The absence of separate ovariectomy-only and dexamethasone-only groups limits the ability to distinguish their individual contributions to bone loss. In addition, the immunohistochemical analyses were based on a semi-quantitative scoring system, which may be subject to observer-related variability. Furthermore, the study focused primarily on histological and structural assessments without detailed molecular validation.

## 5. Conclusions

MK-7 supplementation effectively attenuated bone loss and remodeling imbalance in a rat model of combined ovariectomy- and glucocorticoid-induced osteoporosis. These effects were supported by improvements in bone microarchitecture, modulation of remodeling-related markers, and partial normalization of systemic bone metabolism. Notably, low-dose MK-7 exerted protective effects comparable to high-dose treatment, suggesting that effective modulation of bone remodeling may be achieved within a lower dosing range.

## Data Availability

The original contributions presented in this study are included in the article. Further inquiries can be directed to the corresponding authors.

## Abbreviations

The following abbreviations are used in this manuscript:

MK-7	menaquinone-7
OVX	Ovariectomy
Dex	dexamethasone
